# Coronary artery disease and differential analysis of a valve calcium score by transthoracic echocardiography

**DOI:** 10.1186/cc9451

**Published:** 2011-03-11

**Authors:** JL Iribarren, JJ Jimenez, J Lacalzada, A Barragan, M Brouard, I Laynez

**Affiliations:** 1 Hospital Universitario de Canarias, La Laguna, Spain

## Introduction

Valvular calcification represents a form of atherosclerosis similar to that produced in the wall of the coronary arteries, so that the presence of mitral annular calcification (MAC), aortic valvular sclerosis and aortic root (AVRS) detected by transthoracic echocardiography (TTE) is associated with an increased risk for developing coronary artery disease (CAD). Coronary calcification and intracoronary lesions can be assessed by non-invasive coronary multidetector computed tomography (MDCT). The aim of this study was to determine whether a global valvular calcium score (GVCS) and/or partial (MAC and AVRS) assessed by TTE can predict critical values of calcium at the level of the coronary wall, the Agatston score (AS) and/or the presence of significant coronary lesions detected using MDCT.

## Methods

A prospective cohort of 82 patients with intermediate probability of CAD was referred for MDCT and then a TTE was performed in a blind way to calculate the GVCS and partial (range 0 to 15).

## Results

Mean age 65 ± 13 years, 46 (56.1%) males. The area under the curve (AUC) of AS was 0.69 (95% CI: 0.5 to 0.82), *P *= 0.05. The cut-off value of AS for a higher predictive value to identify the presence of CAD was ≥350 with a sensitivity (S) of 46%, specificity (E) of 86% and a positive predictive value (PPV) and negative predictive value (NPV) of 60% and 78%, respectively. The GVCS value for an AS ≥350 with a higher predictive value was 9. The AUC of GVCS was 0.73 (95% CI: 0.57 to 0.90), *P *= 0.01 so that a GVCS ≥9 predicts the presence of CAD with S = 36%, E = 97%, PPV = 83% and NPV = 79%. Spearman's rho correlation coefficient showed a direct association between AS and GVCS (*r *= 0.29, *P *= 0.03), between AS and MAC (*r *= 0.30, *P *= 0.03) as well as between AS and AVRS (*r *= 0.42, *P *= 0.004). The same coefficient was used to calculate the association between the presence of significant CAD (≥50% stenosis) detected by MDCT and GVCS (*r *= 0.32, *P *= 0.005), MAC (*r *= 0.06, *P *> 0.05) and AVRS (*r *= 0.26, *P *= 0.03). When studying the relationship between single-vessel, double-vessel or triple-vessel CAD and GVCS, MAC and AVRS the following results were obtained respectively: *r *= 0.33 (*P *= 0.004), *r *= 0.06 (*P *> 0.05) and *r *= 0.26 (*P *= 0.03).

## Conclusions

The quantification of valvular calcification using a GVCS by TTE correlates well with the presence of CAD detected by MDCT. This association was stronger when AVRS was used compared with MAC.

